# Activation of acid-sensing ion channels by localized proton transient reveals their role in proton signaling

**DOI:** 10.1038/srep14125

**Published:** 2015-09-15

**Authors:** Wei-Zheng Zeng, Di-Shi Liu, Lu Liu, Liang She, Long-Jun Wu, Tian-Le Xu

**Affiliations:** 1 Discipline of Neuroscience, Department of Anatomy, Histology and Embryology, Collaborative Innovation Center for Brain Science, Shanghai Key Laboratory for Tumor Microenvironment and Inflammation, Shanghai Jiao Tong University School of Medicine, Shanghai 200025, China; 2 Institute of Neuroscience, Chinese Academy of Sciences, Shanghai 200031, China; 3 Department of Cell Biology and Neuroscience, Rutgers University, Piscataway, NJ 08854, USA

## Abstract

Extracellular transients of pH alterations likely mediate signal transduction in the nervous system. Neuronal acid-sensing ion channels (ASICs) act as sensors for extracellular protons, but the mechanism underlying ASIC activation remains largely unknown. Here, we show that, following activation of a light-activated proton pump, *Archaerhodopsin*-3 (Arch), proton transients induced ASIC currents in both neurons and HEK293T cells co-expressing ASIC1a channels. Using chimera proteins that bridge Arch and ASIC1a by a glycine/serine linker, we found that successful coupling occurred within 15 nm distance. Furthermore, two-cell sniffer patch recording revealed that regulated release of protons through either Arch or voltage-gated proton channel Hv1 activated neighbouring cells expressing ASIC1a channels. Finally, computational modelling predicted the peak proton concentration at the intercellular interface to be at pH 6.7, which is acidic enough to activate ASICs *in vivo*. Our results highlight the pathophysiological role of proton signalling in the nervous system.

Acid-sensing ion channels (ASICs), also known as proton-gated cation channels belonging to the degenerin/epithelial sodium channel (DEG/ENaC) family, are highly expressed in the nervous tissue. ASICs are directly activated by extracellular protons and thus exquisitely detect a decrease in pH under both normal and pathological conditions^1^,^2^,^3^,^4^. ASICs have been reported to be required for normal fear responses^5^,^6^,^7^,^8^ and have been implicated in multiple neurological disorders related to tissue acidosis such as epileptic seizures^9^, ischemic stroke^10^,^11^,^12^, and inflammatory pain^13^,^14^,^15^. ASICs desensitize in a relatively quick fashion (within seconds) and their activation requires a significant reduction of pH (EC_50_ = pH 5.0 ∼ 6.5)^1^, raising arguments against the endogenous activation mechanism of ASICs by protons under pathophysiological conditions. Two of these arguments are compelling since during acidosis: (1) pH reductions occur within minutes or hours causing ASICs desensitization^16^; (2) extracellular pH only drops from 7.4 to 6.9, which only partially activates ASICs *in vitro*^5^. Collectively, these concerns point to an unconventional explanation for the activation mechanism of ASICs by protons *in vivo*.

Extracellular pH reduction, as recorded by microelectrodes in brain tissue, has been found to be relatively smaller than it is expected to be^17^. The resolution ability of pH microelectrodes and the strong buffering of the experimental environment may grossly underestimate the local pH changes. Thus, extracellular pH transients *in vivo* during cellular activity could be much steeper, capable of influencing ASICs locally over a limited range. Indeed, the pH fluctuations of the synaptic cleft during synaptic transmission, as measured by a pH-sensitive green fluorescent protein (GFP), were shown to vary from 0.2 to 0.6, due to different stimulation protocols and pH sensors^18^,^19^,^20^. Recent studies from the nematode *Caenorhabditis elegans* showed that protons act as a direct transmitter from intestinal cells to stimulate muscle contraction^21^,^22^. This work demonstrated that protons released from channels/pumps (PBO-4, a putative Na^+^/H^+^ exchanger) activated the pH-sensitive receptors (PBO receptors) and induced muscle contraction during the *C. elegans* defecation motor program. Thus, proton transients especially occurring at areas between tightly apposed cells may play a role in signal transduction. As the mammalian brain expresses proton channels/pumps and pH-sensitive ASICs, we hypothesized that protons can also act as a signal transmitter in the brain^23^. To test this, protons must be released in a highly controllable manner. We utilized the light-activated proton pump, *Archaerhodopsin*-3 (Arch), which is widely used in the optical silencing of neurons^24^,^25^,^26^,^27^. Arch from *Halobacterium sodomense* is a yellow-green light-sensitive opsin that can generate large light-activated proton currents^24^. The excellent kinetics of light-activation (15–85% onset time of 8.8 ± 1.8 ms) and post-light recovery (85–15% offset time of 19.3 ± 2.9 ms) make Arch suitable for providing localized and regulated proton transients^24^.

In the present study, we integrated the optogenetic tool with sniffer patch and performed live-cell imaging to explore the endogenous gating mode of ASICs by localized proton transients. We found that proton transients at the single-cell level could activate ASICs. Furthermore, we found that proton transients from neighbouring cells activate ASICs via the intercellular interface. A mathematical model of diffusion further predicts the proton transients within the intercellular interface. Finally, we demonstrated that protons released from voltage-gated proton channel Hv1 are able to activate ASICs. Taken together, this study underscores the importance of proton sensing and signalling in the brain.

## Results

### Functional coupling between light-activated proton extrusion pump and ASICs

**Figure 1:**
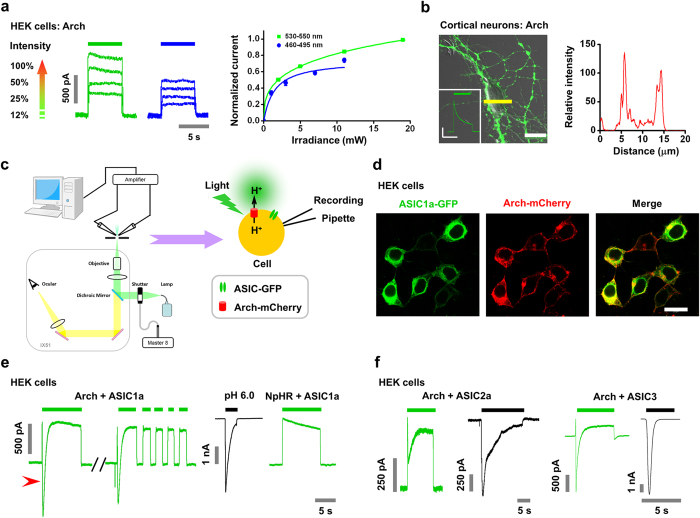
Functional coupling between light-activated proton extrusion pump and ASICs. (**a**) Efficiency of different light stimulations in activating Arch in HEK293T cells. Left, traces of whole-cell recordings from Arch-expressing cell in response to different intensities and wave-ranges of light. Green bar, 530–550 nm; blue bar, 460–495 nm. Right, curves represent single exponential fit; data represent means ± SEM (n = 9). (**b**) Left, confocal image of a mouse cortical neuron expressing Arch-GFP. Scale bar, 10 μm. Insert, trace of Arch activation, illuminated by a 5-s light pulse (green bar, 530–550 nm, irradiance 19 mW); bars, 250 pA, 5 s. Right, a line fluorescence profile (yellow bar in the left image) of Arch-GFP fluorescence demonstrated that Arch-GFP was expressed mainly on cell membranes. (**c**) Left, the *in vitro* light stimulation system. The system is based on an Olympus IX51 upright microscope (gray box). To activate Arch, a green light (530–550 nm) was introduced by a high-pressure mercury lamp. The light was further reflected by a dichroic mirror and focused by the microscope objective to form a restricted light spot on the focal plane (sample). Sample images were captured by CCD camera. Light stimulation with different patterns can be achieved by control of the Master 8 pulse generator. Simultaneously, light-evoked responses were measured by electrophysiology recordings. Right, schematic diagram of optogenetic activation of Arch and ASICs in single cells. (**d**) Confocal fluorescence image of HEK293T cells co-expressing ASIC1a-GFP and Arch-mCherry. Scale bar, 20 μm. (**e**) Left panel: light stimulation (530–550 nm, green bar) of a HEK293T cell that co-expressed ASIC1a-GFP with Arch-mCherry (Arch + ASIC1a) induced ASIC-like inward currents (red arrowhead), which are inactivated following repetitive light stimulation of Arch. Middle panel: pH 6.0 (black bar)-induced current representing the activation of ASIC1a as the positive control in each condition. Right panel: light stimulation of a HEK293T cell that expressed eNpHR3.0-EYFP-2A-ASIC1a (NpHR + ASIC1a) did not induce ASIC-like inward currents (0/15 cells). (**f**) Light stimulation of single HEK293T cells co-expressing ASIC2a-GFP or ASIC3-GFP and Arch-mCherry induced ASIC-like inward currents. The pH 6.0 (black bar)-induced current was the positive control.

To test the idea whether proton transients are able to play a signalling role in mammalian cells as suggested in *C. elegans*^21^,^22^, we took advantage of a highly controllable proton release system by a light-activated proton pump Arch, which is a widely used optogenetic tool for neuronal silencing^24^. A light-activated outward current was successfully recorded in HEK293T cells and cultured cortical neurons overexpressing Arch (Fig. 1a,b). The expression of Arch in neuron is mainly confined to the plasma membrane (Fig. 1b). We next asked whether Arch-generated proton transients activate ASICs in single cells co-expressing ASIC1a, the major form of ASICs in neurons. To this end, we co-expressed ASIC1a and Arch in HEK293T cells (Fig. 1d), and stimulated these cells with different intensities of light and simultaneously recorded light-evoked responses using whole-cell patch-clamp recordings (Fig. 1c). As shown in Fig. 1e, light stimulation not only induced a sustained outward current, but also a transient inward current (Fig. 1e). Two additional neuronal forms of ASICs, ASIC2a and ASIC3, could also be activated by light stimulation of Arch in HEK293T cells co-expressing these proteins (Fig. 1f). The less sensitive ASIC2a showed smaller inward currents in our recordings. Furthermore, this inward current was only observed when light intensity reached a threshold, suggesting that proton transient from Arch must overcome proton buffering barriers before activating ASICs (Supplementary Figure S1). Moreover, the light-induced transient inward current was inactivated following repetitive light-activation of Arch (Supplementary Figure S2). These data collectively suggest that the observed light-induced biphasic currents (Fig. 1e,f) are most likely to result from the activation of Arch, which releases protons and subsequently activate and inactivate ASICs. In support of this notion, no transient inward current was induced in cells co-expressing ASIC1a and *Natronomonas pharaonis* halorhodopsin (NpHR) (Fig. 1e), which hyperpolarizes cells by pumping in chloride ions^28^,^29^. It is unlikely that ASIC1a function was compromised by Arch or NpHR co-expression because stimulation with acid (pH 6.0) induced reliable ASIC1a currents (Fig. 1e).

### Activation of ASICs by Arch-generated proton transients

**Figure 2:**
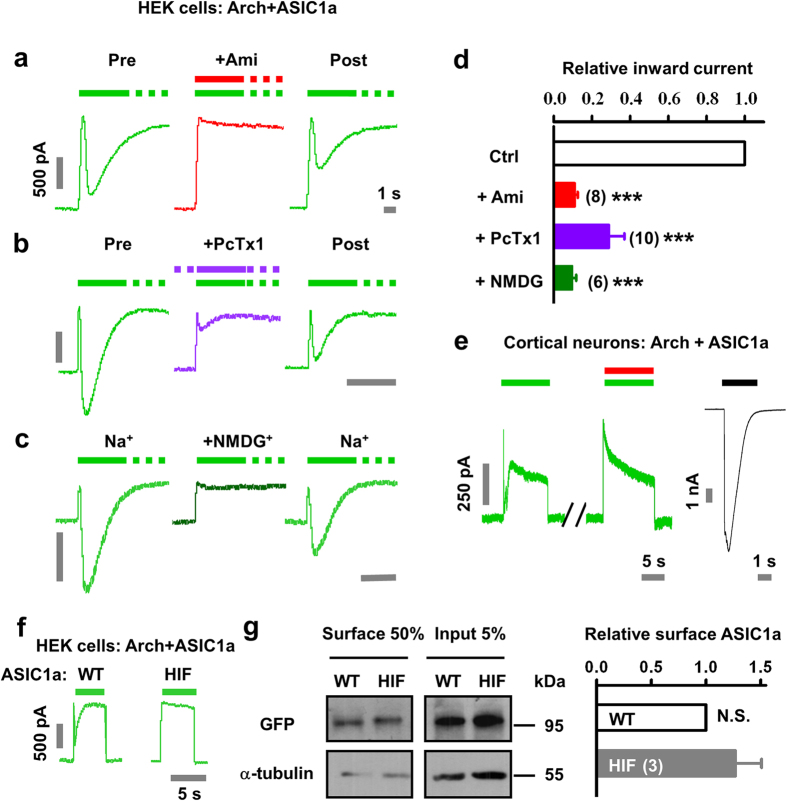
Activation of ASICs by Arch-generated proton transients. (**a**–**d**) Pharmacological profiles of the light (530–550 nm filter)-induced ASIC-like currents in single HEK293T cells co-expressing ASIC1a-GFP and Arch-mCherry. Amiloride (Ami), 100 μM; N-methyl-D-glucamine (NMDG), 150 mM; PcTx1, 100 nM. (**a**–**c)** representative traces. (**d**) Summary for various pharmacological effects. Data represent means ± SEM (***p < 0.001 by paired *t* test, n = 8, 10, and 6 for Ami, NMDG, and PcTx1, respectively). (**e**) Light stimulation of single neurons that co-expressed ASIC1a-GFP and Arch-mCherry induced ASIC-like inward currents. Treatment with Ami (100 μM, red bar) during light stimulation effectively blocked the inward current component. The pH 6.0 (black bar)-induced current was used as a positive control. (**f**) Light failed to induce any detectable inward currents in single HEK293T cells co-expressing the nonconducting ASIC1a mutant ^32^HIF^34–32^AAA^34^ (HIF)-GFP and Arch-mCherry (0/8 cells). (**g**) ASIC1a mutant-GFP has a normal surface expression level compared with ASIC1a WT-GFP control. Surface proteins were determined by surface biotinylation assay. Data represent means ± SEM (N.S., not significant, paired *t* test, n = 3).

To characterize the light-induced inward current further, we applied ASIC channel blockers, depleted the extracellular sodium ion concentration, and tested the nonconducting ASIC1a mutant (^32^HIF^34–32^AAA^34^, HIF)^30^. First, both the pan-ASICs blocker amiloride (Ami) and ASIC1a channel-specific blocker psalmotoxin 1 (PcTX1)^31^ inhibited the light-induced inward current in HEK293T cells co-expressing ASIC1a and Arch (Fig. 2a,b,d). The light-induced inward current was also blocked by pan-ASICs blocker Ami in cultured mouse cortical neurons co-expressing Arch and ASIC1a (Fig. 2e). Second, the substitution of extracellular sodium ions with channel impermeable *N*-methyl-D-glucamine^32^ also abolished the light-induced inward current (Fig. 2c,d). Third, in HEK293T cells co-expressing Arch and the nonconducting ASIC1a mutant, no inward peak current was detected (0/8 cells; Fig. 2f). The surface expression level of HIF mutant, as evaluated by biotinylation assay, was comparable to that of the wild type (WT) (HIF, 1.27 ± 0.24 times than WT, *p* = 0.37, paired *t* test, n = 3), suggesting that the absence of inward current was not due to inefficient delivery of mutant channels to the plasma membrane (Fig. 2g). Taken together, these data support that proton transients achieved by light stimulation of Arch activates co-expressed ASICs in HEK cells or neurons at the single-cell level.

### Characterization of Arch-ASIC1a interaction in single cells

**Figure 3:**
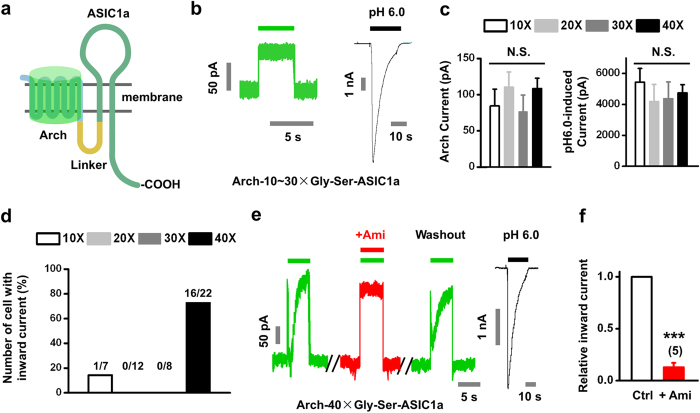
Characterization of Arch-ASIC1a interaction in single cells. (**a**) Schematic diagram of Arch-ASIC1a chimera. A flexible linker (marked with yellow) of GS repeats is constituted by 10, 20, 30 or 40 amino acids. (**b**) Only Arch but not an ASIC-like current was observed when the linker was varied between 10 and 30 amino acids. The pH 6.0 (black bar)-induced current was used as a positive control. (**c**) Statistical results of light-activated Arch (left) and pH 6.0-induced ASIC1a currents (right) with various length of linker as indicated. Data represent means ± SEM (N.S., not significant of each two group, by unpaired *t* test, n = 7, 12, 8, 22 for 10, 20, 30 and 40 amino acids of linker, respectively). (**d**) The percentage of cells showing light-induced inward current in each group. (**e**,**f**), Light (530–550 nm filter, green bar)-induced ASIC1a activation by Arch only when the linker was 40 amino acids long (Arch-40 × Gly-Ser-ASIC1a). Ami, 100 μM (red bar). The pH 6.0 (black bar)-induced current was used as a positive control. (**f**) Summary data from (**e**) Ctrl, control. Data represent means ± SEM (***p < 0.001 by paired *t* test, n = 5).

To assess quantitatively the activation distance of ASIC1a by Arch, we constructed a series of chimera proteins that bridge the C-termini of Arch and N-termini of ASIC1a by flexible glycine/serine (GS) linker, ranging from 10 to 40 amino acids (Fig. 3a). The GS linker is widely used in antibody engineering to join two peptides together^33^,^34^. We transfected the Arch-ASIC1a chimera into HEK293T cells and measured the light-induced currents using whole-cell recordings. First, we validated the function of Arch and ASIC1a in the chimera. We found that the light-induced Arch current was smaller with fusion proteins than when expressed alone, which may be due to the stiffness of the linker (Fig. 3b). However, chimeric Arch showed no significant difference in currents between each group with different linkers (10 × linker, 84.4 ± 23.2 pA, n = 7; 20 × linker, 110.4 ± 21.1 pA, n = 12; 30 × linker, 76.6 ± 23.0 pA, n = 8; 40 × linker, 108.3 ± 14.6 pA, n = 22; N.S., not significant for each two groups, unpaired *t* test; Fig.3b,c). The function of ASIC was not compromised by its fusion with Arch (Fig. 3b,c). In addition, pH 6.0-induced ASIC1a currents remained intact in each chimera group (10 × linker, 5430.9 ± 891.0 pA, n = 7; 20 × linker, 4188.0 ± 1099.0 pA, n = 12; 30 × linker, 4366.2 ± 1090.0 pA, n = 8; 40 × linker, 4743.4 ± 535.8 pA, n = 22; N.S., not significant for each two groups, unpaired *t* test; Fig. 3b,c). We then tested the likelihood of light-induced inward currents in each group. In the linker varying from 10 to 30 amino acids groups, we barely recorded light-induced inward currents (14.3%, 1 out of 7 cells; 0 out of 12 cells; 0 out of 8 cells, respectively; Fig. 3d). However, when the linker was expanded to 40 amino acids, the chance of invoking currents following light stimulation was significantly increased (72.7%, 16 out of 22 cells; Fig. 3d). Again, the light-induced inward current is mediated by ASIC1a as it can be abolished by treatment with Ami in these cells (0.13 ± 0.04 times that of the control; *p* < 0.001 by paired *t* test; n = 5; Fig. 3e,f). Although the estimated length of a GS linker of 40 amino acids is around 15 nm, the apparent distance between two proteins may be much shorter owing to the flexibility of the linker^35^. Nevertheless, these data are consistent with the notion that the light-induced ASIC currents depend on protons released from functional Arch.

### Activation of ASIC1a channels by localized proton transients at intercellular interface

**Figure 4:**
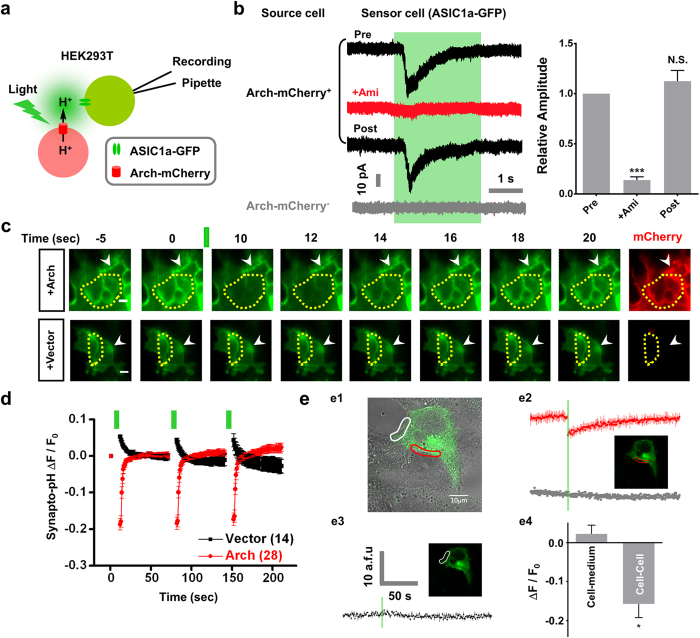
Activation of ASIC1a channels by localized proton transients at intercellular interface. (**a**,**b**) Proton transients activate ASIC1a intercellularly. (**a**) Schematic diagram of a two-cell sniffer patch using Arch-mCherry (source cell) and ASIC1a-GFP-expressing (sensor cell) HEK293T cells. (**b**) Results for two-cell sniffer patch from a sensor cell. Left: representative traces. Black traces showed light (530–550 nm, green region)-induced inward current (Pre) when the source cell expressed Arch-mCherry (mCherry^+^). The inward current was inhibited by Ami (100 μM) and recovered after 3 min wash (Post). No response was recorded when the source cell did not express Arch-mCherry (mCherry^−^, grey trace). Right: Statistical results from black traces. Inward currents were normalized relative to Pre group. Data represent means ± SEM (***p < 0.001; N.S., not significant; by paired *t* test, n = 6). (**c**) Live imaging of light-induced extracellular acidification by Arch within 25 seconds. The pHluorin fluorescence from HEK293T cells co-expressing synapto-pHluorin and vector or Arch-mCherry was monitored following 510–560 nm light stimulation (green bars, 10 s duration, 60 s interval). Arrowheads indicate the cell-medium surfaces, while the intercellular interface is surrounded by yellow dotted lines. Scale bar, 10 μm. (**d**) Summary data of (**c**). Data represent means ± SEM (n = 14 and 28 for vector and Arch, respectively). (**e**) Light-induced extracellular acidification was evident at the intercellular interface. (**e1**) HEK293T cells co-expressing synapto-pHluorin and Arch-mCherry were stimulated by green laser (555 nm) from confocal microscope at different region of interests (ROI). The cell-medium and intercellular interfaces are circled by a white and red line, respectively. (**e2**) pHluorin fluorescence changes within intercellular interface when stimulated. Red trace, HEK293T cells co-expressing synapto-pHluorin and Arch-mCherry. Grey trace, cells co-expressing vector and Arch-mCherry were used as a negative control. (**e3**) pHluorin fluorescence changes within cell-medium area when stimulated. Black trace, cells were co-expressing synapto-pHluorin and Arch-mCherry. (**e4**) Statistical results of pHluorin fluorescence changes at different ROI after light stimulation. Cells were co-expressing synapto-pHluorin and Arch-mCherry. Data represent means ± SEM (*p < 0.05; by unpaired *t* test, n = 12).

Given that the proton source and sensor could be located in different cells *in vivo*^21^, we asked whether proton transients activate ASICs intercellularly. To this end, we used two-cell sniffer patch in source cells overexpressing Arch-mCherry and sensor cells overexpressing ASIC1a-GFP (Fig. 4a). The sensor cell was whole-cell patch-clamped to monitor the ASIC current, which is likely to be activated by protons released from its nearby source cells stimulated by light. Here, we recorded two forms of sensor cells, one bound by a mCherry fluorescence positive (Arch-mCherry^+^) source cell, and another bound by a mCherry fluorescence negative (Arch-mCherry^−^) cell. Upon light stimulation, proton release through Arch activation was detected as an inward current mediated by ASIC1a in the sensor cells adjacent to the Arch-mCherry^+^ source cell (Fig. 4b). The inward current was inhibited by Ami and was reproducible after Ami washout, confirming the ASIC1a activation by source cell (+Ami, 13.8 ± 3.3% compared with Pre, p < 0.001; Post, 112.4 ± 10.7% compared with Pre, not significant; paired *t* test, n = 6; Fig. 4b). In contrast, the sensor cells close to the Arch-mCherry^−^ source cells showed no response upon light stimulation (grey trace, Fig. 4b). We observed a ~500 ms delay between the activation of Arch on source cell and the appearance of ASIC1a currents on sensor cell (Fig. 4b), which differed from that observed in single cells co-expressing Arch and ASIC1a (Figs 1, 2, 3). The delay is presumably due to the proton diffusion from the source to sensor cells, and thus is likely to be determined by the distance between two cells and proton buffering capacity of the culture solution. Nevertheless, these data indicate that proton transients released from a neighbouring cell are able to activate ASIC1a channels in adjacent cells.

To directly visualize Arch-generated acidification at the intercellular interface, we next utilized various molecular and live-cell imaging tools. pHluorin is a pH-sensitive GFP mutant, the absorbance of which decreases as the pH is lowered (has a pKa of ~7.1)^36^. The fusion of pHluorin with the luminal C-terminus of vesicle-associated membrane protein (VAMP), which is also called synapto-pHluorin, has been widely used to investigate the dynamics of exocytosis and vesicle recycling at the presynaptic terminal^36^,^37^. To monitor directly Arch-induced extracellular acidification, we monitored the surface fluorescence of synapto-pHluorin to characterize the pH changes upon light stimulation. Indeed, full-field light stimulation induced a significant pH drop, as indicated by reduction in pHluorin fluorescence (peak fluorescence changes: 0.19 ± 0.02 ΔF/F_0_) in HEK293T cells co-expressing Arch and synapto-pHluorin (Fig. 4c,d). A slower alkalinization was observed following acidification, which is presumably due to the activation of Arch^24^ and the broad expression of synapto-pHluorin in both cytoplasm and plasma membrane. In contrast, the pHluorin fluorescence remained unchanged (peak fluorescence changes: 0.05 ± 0.003 ΔF/F_0_) in the HEK293T group co-expressing Arch with vector control (Fig. 4c,d). Interestingly, we observed that light-induced pHluorin fluorescence reduced more significantly at the intercellular interface (indicated by yellow dotted lines, Fig. 4c) than at the cell-medium interface (indicated by arrowheads, Fig. 4c). To further characterize the dynamics of proton transients at higher spatial and temporal resolution, we performed confocal imaging in live HEK293T cells co-expressing Arch and synapto-pHluorin and stimulated specific region of interests (ROI). The cell-medium and cell-cell interface area were indicated by a white and red circle, respectively (Fig. 4e1). Indeed, light stimulation substantially reduced pHluorin fluorescence at the cell-cell ROI (peak fluorescence changes: 0.16 ± 0.04 ΔF/F_0_; Fig. 4e2, red trace; Fig. 4e4) but not in the cell-medium ROI (peak fluorescence changes: 0.02 ± 0.02 ΔF/F_0_; Fig. 4e3, black traces; Fig. 4e4). As a negative control, light stimulation did not change fluorescence in HEK293T cells co-expressing vector control and synapto-pHluorin (Fig. 4e2, grey trace). These data indicate that proton transients formed at the intercellular interface are efficient for ASIC activation and proton signalling. We predicted that such proton signalling could also happen *in vivo* owing to the concentrated proton diffusion in the narrow space *in situ*, which has been shown by recent findings at the synaptic cleft and muscle-intestinal interface^18^,^19^,^20^,^21^.

### Computational modelling predicts proton-sensing capacity of ASICs *in vivo*

**Figure 5:**
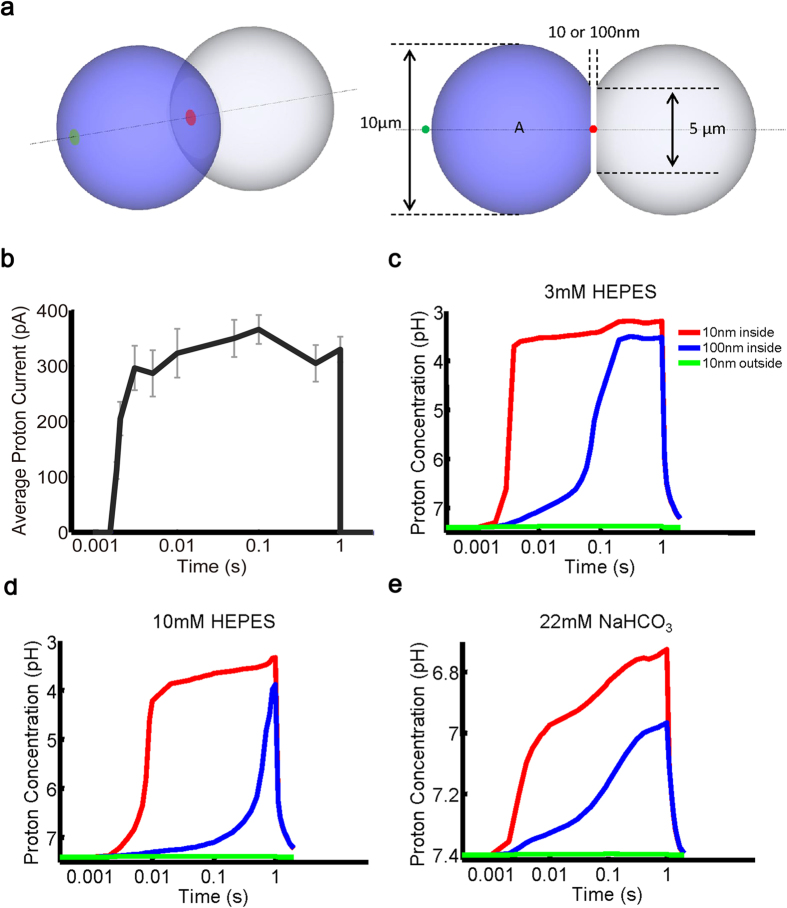
Computational simulation of proton diffusion predicts the activation possibility of ASICs *in vivo*. (**a**) Perspective and side view of the diffusion model. Geometry of a single HEK293T cell is modelled by a spheroid with 10 μM diameter. The detection points of the extracellular pH changes after proton release from the source cell (blue spheroid) are divided into intercellular (between the source cell and sensor cell, red disc) and cell-medium interface (green disc), which are simulated differently. (**b**) The proton current of single HEK293T cells expressing Arch was measured by whole-cell recording. The illumination of light (530–550 nm filter) was controlled by a high-speed shutter, which opens from 1.5 ms to 1 s. For different data points, n value varies from 3 to 7. (**c–e**) Computational simulation results in 3 mM HEPES (**c**), 10 mM HEPES (**d**), and 5% CO_2_/22 mM NaHCO_3_ (**e**) extracellular solution. Shown are extracellular pH changes after the activation of Arch by light. The red and blue curves are pH changes in the red disc, which represents the interface between the source cell and sensor cell and varied from 10 to 100 nm, respectively. The green curve is the pH changes of the green disc, which represents the cell-medium interface and is 10 nm from the source cell.

To evaluate the activation conditions of ASICs *in vivo*, we utilized a computational diffusion model. The geometrical constraint of a HEK293T cell was considered to be a 10-μm diameter spheroid. An intercellular interface ranging from 10 to 100 nm was used to mimic the distance of a gap junction and chemical synapse cleft^38^ (Fig. 5a). To detect the diffused protons, two detection points were set at the intercellular (red disc) and cell-medium (green disc) interfaces (Fig. 5a). The diffusion of protons in solution was restricted by the following factors: (1) the rate of a proton released from a single cell; (2) the anions that determine the buffering ability of a solution; and (3) diffusion rates of the molecules (see Methods). To estimate the proton release rate, Arch-generated whole-cell currents which reflect proton efflux, were measured by electrophysiological recordings in HEK 293T cells (Fig. 5b). Next, we altered the concentration and species of anions during the computer calculation to estimate their influence on proton transmission. In our computational model, proton transients were predicted to appear in the intercellular interface, and was largely dependent on the buffering ability of solution and the distance from the source cell (Fig. 5c–e, red and blue lines). In contrast, pH changes in the cell-medium interface were negligible (Fig. 5c–e, green line). Although the diffusion coefficient outside the cells was the same, there was an obvious difference in proton concentration between the intercellular and cell-medium interfaces (Fig. 5c–e). Therefore, the spatial distribution between source and sensor cells has significant influence on proton concentration around the source cell. Proton transients were then calculated in HEPES buffer or CO_2_/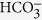 buffer. We found that the peak proton concentration was reached at a pH 3.2 in the centre of the intercellular interface (the distance between two cells is 10 nm) in the presence of 3 mM HEPES solution. Interestingly, the physiological CO_2_/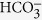 buffer showed stronger proton buffering ability than HEPES, and peak proton concentration was reached at pH 6.7 in CO_2_/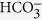 buffer (Fig. 5e). In summary, the computational model confirms the existence of proton transients in the intercellular interface, suggesting the possibility of ASIC activation via proton signalling *in vivo*.

### Functional coupling between proton extrusion channel and ASIC1a channel

**Figure 6:**
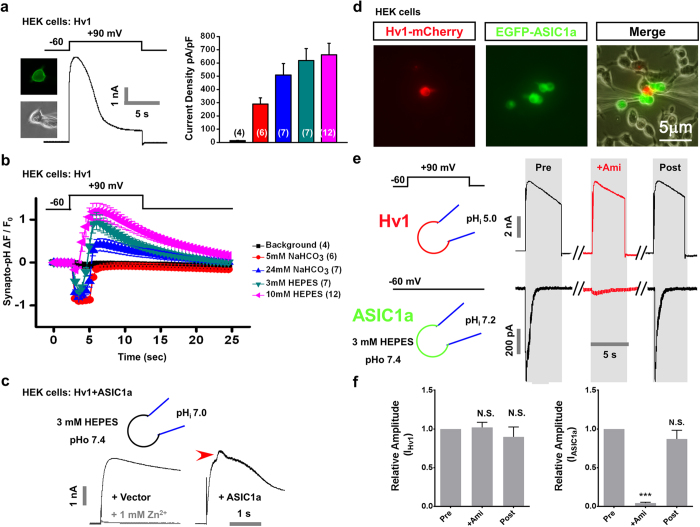
Functional coupling between Hv1 and ASIC1a channels. (**a**) Left, Hv1 currents were recorded and extracellular acidification was monitored in HEK293T cells co-expressing Hv1-mCherry and synapto-pHluorin. Hv1 was activated by membrane depolarization from −60 to +90 mV. The recording pipette was filled with internal solution at pH 5.0. The external solution was buffered with 10 mM HEPES at pH 7.4.A representative trace of Hv1 activation is shown. Insert, representative images of pHluorin fluorescence and DIC. The recording pipette was indicated in DIC image. Right, pooled data of current density measurements under different extracellular pH buffers. (**b**) pHluorin fluorescence changes related to Hv1 activation under different extracellular pH buffers as indicated. (**c**) Coupling between Hv1 and ASIC1a in HEK293T cells. The pH of external and internal recording solutions is indicated. Left, representative traces of Hv1 current when HEK293T cells were cotransfected with vector control (black trace). Application of 1 mM Zn^2+^ abolished the Hv1 channel activity (grey trace). Right, ASIC1a was co-expressed with Hv1. The ASIC-like current is indicated by a red arrowhead. Similar results were obtained from three other cells. (**d–f**) Two-cell sniffer patch showing that protons released from the source cell via Hv1 channel activate ASIC1a in the sensor cell. (**d**) Representative images illustrating double patch configurations in HEK293T cells that co-express Hv1-mCherry and EGFP-ASIC1a. (**e**) Representative traces of Hv1 and ASIC1a responses before (Pre), during (+Ami, 100 μM) and after washout (Post) of Ami. Upper panel, induction of Hv1 current by depolarization from −60 to +90 mV. Lower panel, current responses of a sensor cell expressing ASIC1a. The cell was holding at −60 mV. (**f**) Pooled data of Ami effects on Hv1 (left) and ASIC1a (right) currents, respectively. Data represent means ± SEM (N.S., not significant; ***p < 0.001, +Ami *vs* Pre; paired *t* test, n = 6).

Voltage-gated proton channel Hv1 extrudes protons in macrophages and microglia upon cell membrane depolarization, which regulates intracellular pH and NADPH oxidase-dependent generation of reactive oxygen species (ROS)^39^. We asked whether proton transients generated from Hv1 activation are able to activate ASICs. We first tested the pH regulation ability of Hv1 upon activation in HEK293T cells co-expressing Hv1 and synapto-pHluorin. To amplify the Hv1 response, the pH of the internal recording solution was adjusted to 5.0 (Fig. 6a,b). Another advantage for lowering the pH inside the cell is that it can eliminate the pHluorin fluorescence and thus only pHluorin inserted into the cell surface could show a signal. When Hv1 was activated by depolarizing HEK293T cells (from—60 to + 90 mV, Fig. 6a), an outward current was recorded for various pH buffering systems (background control, 12.5 ± 3.4 pA/pF, n = 4; 5 mM NaHCO_3_, 289.8 ± 46.3 pA/pF, n = 6; 24 mM NaHCO_3_, 509.2 ± 86.5 pA/pF, n = 7; 3 mM HEPES, 619.4 ± 90.4 pA/pF, n = 7; 10 mM HEPES, 662.4 ± 87.8 pA/pF, n = 12; Fig. 6a, right panel. The pH buffers are the same as in Fig. 6b). The current increased as the extracellular buffering capacity grows stronger, reflecting the proton-dependence of Hv1 activation^40^. It is obvious that stronger buffer reduced extracellular free protons and thus created a higher proton gradient across the plasma membrane, resulting in more proton extrusion through activated Hv1 channels. The pHluorin fluorescence was simultaneously monitored and revealed a biphasic pHluorin fluorescence change. At the start, the cell surface fluorescence signal decreased rapidly owing to the drop in the extracellular pH (pH_o_), which was likely to be caused by Hv1 opening and proton extrusion. Following prolonged depolarization, sustained activation of Hv1 led to an increase in the intracellular pH (pH_i_), gradually drawing back the fluorescence signal (Fig. 6b). These results indicate that Hv1 activation is able to provide a regulated proton transient.

We then tested whether Hv1-generated proton transients can also activate ASICs as Arch does (Fig. 1e). We co-expressed Hv1 and ASIC1a in single HEK 293T cells. For recording ASIC1a currents, we adjusted the pH_i_ to 7.0 (indicated in Fig. 6c) since intracellular acidification inhibited the ASIC current^41^. We observed a large outward current, which was fully abolished by Hv1 channel blocker Zn^2+^ in Hv1-transfecting cells (Fig. 6c). When ASIC1a was co-transfected, an additional outward current was recorded. The rapid deactivation current was reminiscent of ASIC-like responses which are outward with the holding potential at +90 mV and 120 mM NaCl inside the cell (Fig. 6c). However, such ASIC1a responses were largely compromised by the much larger Hv1 currents in a single cell during membrane depolarization. To better demonstrate Hv1-ASIC1a coupling, we performed two-cell sniffer patch in cells expressing Hv1 and ASIC1a separately (Fig. 6d–f). HEK293T cells expressing Hv1-mCherry (red) and ASIC1a-EGFP (green) were patched by double recording pipettes (Fig. 6d). Depolarization from −60 to +90 mV induced an outward current in Hv1-mCherry expressing cells. Meanwhile, we recorded a significant ASIC-like current in the neighbouring ASIC1a-EGFP cells (Fig. 6e). The inward current was reversibly inhibited by the ASIC blocker (+Ami, 100 μM, 4.5 ± 0.9% compared to Pre group, *p *< 0.001; Post, 87.1 ± 11.3% compared to Pre group, not significant; paired *t* test, n = 6; Fig. 6e,f), reflecting the pharmacological profile of ASICs. Ami showed no influence on the Hv1 current itself in red cell (+Ami, 102 ± 6.6% compared to Pre group, not significant; Post, 90 ± 12.7% compared to Pre group, not significant; paired *t* test, n = 6; Fig. 6e,f). Meanwhile, we tested various depolarizations to activate Hv1 proton channel and found that proton currents induced by a more physiological depolarization, for example, from −60 to +10 mV is sufficient to activate the ASIC1a channels expressed in neighboring cells (Supplementary Figure S3). These results demonstrated that protons from source cell expressing Hv1 are able to activate ASIC1a in the sensor cell. Together, our data provides a proof-of-principle that proton signalling could be mediated by Hv1-ASIC1a coupling and Hv1 proton channel could be a potential proton source that may activate ASICs *in vivo*.

## Discussion

The extracellular proton signalling in mammalian cells poses a novel aspect for cellular communication. As an exquisite proton sensor in the nervous system^1^, ASICs play an important role in proton signal transduction^23^. However, it is unclear whether protons are released in a physiologically relevant manner to activate ASICs and mediate intercellular communication, although accumulating evidence implicates the endogenous activation of ASICs by protons in the nervous system^20^. Recently, Li *et al.* took advantage of light-activated proton pumps (Arch) and demonstrated that ASICs can be activated by a localized proton source^42^. First, the present study confirmed that ASIC currents could be recorded after the activation of Arch at the single-cell level (Figs 1 and 2). Furthermore, we showed that such functional coupling could be achieved by co-expressing ASICs with Arch or directly coupling these proteins as a chimera by molecular cloning (Fig. 3). Second, our data demonstrated that proton transients from the neighbouring cell activated ASIC1a and were more efficiently transmitted via the intercellular interface (Fig. 4). Third, we utilized a computational diffusion model to predict the proton concentration from a source cell at any spatial point and estimated the possible criteria for the activation of ASICs *in vivo* (Fig. 5). Finally, we showed that protons released by Hv1 proton channels are able to activate ASICs (Fig. 6).

Protons have a fundamental role in organisms, which is to maintain the structure and function of proteins. Moreover, protonation-deprotonation events dictate the charge of biological surfaces and are an integral part of many metabolic reactions^43^. It is usually assumed that protons influence the intracellular pH homeostasis and metabolism^43^, whereas their extracellular role remains unclear. Recent work in *C. elegans* showed that extracellular protons act as transmitters^21^,^22^. The defecation rhythm of *C. elegans* was driven by proton flux in addition to the calcium oscillation. Intestinal cells periodically extrude protons across their basolateral surface through the Na^+^/H^+^ exchanger (NHE) PBO-4 (also known as NHX-7; the ortholog of mammalian NHE1), which results in acidification of the extracellular space and activation of proton-gated cation channels (composed of PBO-5 and PBO-6 subunits) located in the adjacent muscle cells, thereby inducing their contraction during the defecation cycle of the nematode^21^,^22^. These observations raise the possibility that protons secreted or released at synapses might act as primary intercellular messengers in the modulation of pH-sensitive proteins such as ASICs. To further support this, recent studies have demonstrated the generality of protonergic neural transmission in the synaptic cleft from inner ear, retina, and amygdala^18^,^19^,^20^.

The endogenous activation of ASICs is modulated by the following three factors. First, abundant protons must be released from the source in a regulated way. It is believed that biological protons are generated by two basic mechanisms: the *de novo* production of acid species and the net transmembrane fluxes of protons or their equivalents. The former arises mostly from energy metabolism, develops slowly, and is long-lasting (such as metabolic acidosis). Conversely, the latter arises from the activation of proton-permeable ion channels or pumps^23^. While global changes in intracellular or extracellular pH are likely to be harmful and are unlikely to serve as signals, transient and localized pH changes from ion channels/pumps could play such a role. Second, an intercellular interface must exist to transmit protons. In contrast to the slow diffusion rate in the cytosol (diffusion constant 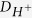 = 0.4–2.2 × 10^−12^ m^2^/s)^43^, protons diffuse two orders of magnitude faster in water (diffusion constant 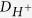 = 9.3 × 10^−9^ m^2^/s)^44^, which provides the possibility of signal transduction between cells. In multicellular organisms, cells are always interacting with the physiological fluid and are surrounded by each other. Intercellular interfaces, such as those found at neuronal synapses and neuron-glia interactions, are fundamental structures for proton signalling^38^. These structures, ranging from 3 to 100 nm, contain intensive signalling molecules such as ion channels, neurotransmitters, and cell adhesion molecules^38^,^45^,^46^. Since proton diffusion is restricted in the limited space, proton concentration within the intercellular interface is expected to be much higher than in the cell medium. A previous study has also shown that carbonic anhydrase facilitated intracellular proton mobility 5.8 times faster in CO_2_/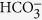 (carbonic) buffer^47^, which was not included in our model (Fig. 5). Given that extracellular carbonic anhydrase is active *in vivo*^48^, it is reasonable to speculate that the apparent proton mobility in the carbonic buffer should be faster than in the computer simulation. Third, like the N-methy-D-aspartate receptors on postsynaptic densities or nicotinic acetylcholine receptors on the neuromuscular junction^49^, we hypothesize that ASICs localized in the sensor cell should be highly concentrated to receive the upstream proton signal and generate downstream signal cascades. However, there is still no conclusive evidence for the expression pattern of ASICs in neurons or other cell types. In a previous study, we utilized an extracellular haemagglutinin (HA)-tagged ASIC1a to visualize its expression and found that the distribution of ASIC1a^50^, 2a and 3 (Zeng *et al.* unpublished observations) on the plasma membrane is highly restricted and organized. These findings imply that ASICs could effectively sense the endogenous proton transients by forming puncta-like structure. Taken together, we suspect that the synapse cleft is an attractive target to study the contribution of ASICs in proton signalling, although postsynaptic ASIC currents have not been detected in cultured hippocampal neurons^51^,^52^. Interestingly, a recent study by Du and colleagues demonstrated the existence of postsynaptic ASIC current activated by synaptic transmission in lateral amygdala neurons. The ASIC-mediated proton signalling was further shown to be critical for amygdala-dependent learning and memory^20^.

Here we demonstrate that proton transients from neighbouring source cells can activate ASICs in sensor cells via their intercellular interface *in vitro* (Fig. 4). It is important to explore the source of the protons that activate ASICs *in vivo*. Voltage-gated proton channel Hv1 and Na^+^/H^+^ exchangers have emerged as strong candidates for generating proton transients. Hv1 is a voltage-gated proton channel with specific expression in immune cells and has high selectivity to protons^53^,^54^. The most established function of Hv1 is the production of ROS by phagocytic cells. Interestingly, Hv1 is highly expressed in brain microglia and is required for NADPH oxidase (NOX)-dependent ROS generation^55^. Microglia continuously interact with surrounding neurons by extending their ramified processes^56^,^57^. The neuron-microglia interaction has been found to be actively involved in regulating neuronal activity^58^,^59^ and the brain immune response during inflammation^60^,^61^. Mice lacking Hv1 are resistant to NOX-mediated neuronal death and ischemic stroke^55^. Coincidentally, genetic deletion of ASIC1a extenuates neuronal death in ischemic models^11^,^12^. As a proof of concept, we successfully recorded an ASIC1a response after Hv1 activation in cell lines (Fig. 6c–f). Since the Hv1 proton channel is expressed mainly in microglia while ASICs in neurons, our results hint that the depolarization from microglia could propagate to neighbouring neurons by Hv1-ASICs crosstalk. Future studies are needed to study whether the signalling of Hv1-ASICs coupling exists both in the healthy and diseased brain. NHEs have been first speculated as the endogenous agonist of ASICs^21^. NHE1 is the most ubiquitously expressed isoform on the plasma membrane and regulates a number of cell behaviours, including adhesion, shape determination, migration, and proliferation^62^. An ortholog of mammalian NHE1 in *C. elegans* was shown to mediate proton signalling and induce the defecation cycle^43^, supporting the idea that localized protons can act as signalling molecules. Since the results of a previous study implied that the expression pattern of ASIC1a and NHE1 do not overlap in neurons^63^, the coupling between NHE1 and ASICs, if it occurs, seems to be highly dynamic.

In conclusion, our data indicate that proton transients in single and paired-cell mode can activate ASICs. We also show that cell-cell adhesion provides the transmission environment for proton signalling. We propose that physiological proton transients from ion channels/pumps can activate ASICs. These results reveal a contribution of ASICs in endogenous proton signalling, which may be particularly important under pathological conditions such as ischemia and seizure, in which proton signalling is augmented.

## Methods

### Primary neuron cultures and mammalian cell lines

All animal procedures were carried out in accordance with the guidelines for the Care and Use of Laboratory Animals of Shanghai Jiao Tong University School of Medicine (Policy Number DLAS-MP-ANIM.01–05) and approved by the Institutional Animal Care and Use Committee (Department of Laboratory Animal Science (DLAS), Shanghai Jiao Tong University School of Medicine). Dissociated cortical neurons were prepared and maintained as described previously^14^. Briefly, cerebral cortices from 16-day-old embryonic C57BL/6J mice were dissected and dissociated using 0.05% trypsin in D-Hank’s solution for 10 minutes. Cells were plated (3 × 10^5^ cells/ml for electrophysiology) on poly-D-lysine (Sigma)-coated cover glasses or 6-cm dishes (Corning). Cultures were maintained in Neurobasal medium containing 2% B27 and 1% Glutamax supplements (Invitrogen) at 37 °C for 14–16 days prior to experiments. Human Embryonic Kidney (HEK) 293T cells were grown in DMEM medium with 10% foetal bovine serum and penicillin/streptomycin (Invitrogen).

### Transfection and plasmids

HEK293T cells or cultured cortical neurons were transfected with an appropriate amount of DNA (3.5 μg DNA for electrophysiology and immunocytochemistry; 8 μg DNA for biochemistry) mixed with HilyMax liposome transfection reagent (Dojindo Laboratories). Equal amounts of each plasmid were used for cotransfection. Proteins were allowed to express for 24 to 48 hours prior to experiments. The cDNA of human ASIC1a was cloned into the pEGFPC3 vector (Promega). For the GFP-ASIC1a plasmid, GFP was linked at the N-terminus of human ASIC1a. Arch plasmid was kindly provided by Prof. Edward S. Boyden (Massachusetts Institute of Technology). Synapto-pHluorin was kindly provided by Prof. Gero Miesenböck (University of Oxford).

### Optogenetic setup and pHluorin imaging

The system was built on an inverted commercial microscope (IX51, Olympus, Japan). The main optical path is illustrated in Fig. 1c. In the light stimulation system, the beam from a 120 W mercury arc lamp (X-Cite 120Q System, Lumen Dynamics, Canada) was coupled into a light guide (LLG, Lumen Dynamics, Canada) and filtered through a 530–550 nm filter (U-MNG2 fluorescence mirror unit, Olympus, Japan). To accurately regulate the time of light stimulation, a shutter (LS6, Uniblitz, USA) controlled by a pulse generator (Master 8, A.M.P.I, Israel) was equipped at the entrance of light to the microscope. After further passing through the objective (LUCPLFLN 40×, Olympus, Japan), the laser beam was focused onto the focal plate to yield a restrictive spot (10 mm in diameter, 19 ± 1 mW).

The pHluorin signal was captured by an inverted commercial microscope (TE2000E, Nikon, Japan) equipped with a 40 × Plan Apochromat, oil-immersion objective. pHluorin was excited with 490–500 nm light, while Arch was activated with 510–560 nm light from a mercury arc lamp (Intensilight, Nikon, Japan) with a 10-s exposure time at 60-s intervals. Images were recorded with a 16-bit cooled-CCD camera (Roper Cascade 512B, Photometrics, USA). All acquired images were processed by ImageJ (National Institutes of Health) software.

### Electrophysiology

Whole-cell recordings from cultured neurons (14–18 DIV) and cell cultures were performed by Axopatch 200B with Digidata 1440A and the pClamp10 software (Molecular Devices). Whole-cell pipettes (4–6 MΩ) were used with internal solution comprising (in mM): 120 KCl, 30 NaCl, 0.5 CaCl_2_, 1 MgCl_2_, 5 EGTA, 2 MgATP, and 10 HEPES (pH 7.2). The HEPES-buffered normal extracellular solution contained (in mM): 150 NaCl, 5 KCl, 1 MgCl_2_, 2 CaCl_2_, 10 glucose and 10 HEPES (buffered to various pH values with NaOH or HCl). For Hv1 recording and pHluorin imaging (Fig. 6a,b), pipettes were filled with (in mM) 120 CsCl and 100 MES (pH 5.0). For recording the functional coupling between ASIC1a and Hv1 in single cells (Fig. 6c), pipettes were filled with (in mM) 120 NaCl and 10 HEPES (pH 7.0). For two-cell sniffer patch recording of Hv1 and ASIC1a response, pipettes were filled with (in mM) 120 CsCl, 100 MES (pH 5.0) and 120 KCl, 10 HEPES (pH 7.2), respectively. Currents were recorded with cells voltage-clamped at −60 mV.

### Modelling using the diffusion equation

The diffusion equation was used to estimate the proton concentration released from a single HEK293T cell at any extracellular domain, including the intercellular and cell-medium interface. The geometrical constraints of a HEK293T cell were considered as a uniform spheroid with a 10-μm diameter. Light-induced activation of Arch pumped protons out of the cell and acidified the extracellular region. Hence, three factors that affect the extracellular pH were included in the simulation:

(1) The rate of proton released from a single cell. It was measured via whole-cell recording of Arch-expressing HEK293T cells that were activated by light stimulation (530–550 nm filter) from 1.5 ms to 1 s (Fig. 5b). The proton flux was assumed to be uniformly distributed on the spheroid surface of the 10-μm diameter.

(2) The chemical equilibrium of proton and the anions in the solution (OH^−^, HEPES^−^ or HCO_3_^−^). Three types of extracellular solutions with different buffering ability were used in the computer simulation (in mM): (a) HEPES-buffered normal extracellular solution (150 NaCl, 5 KCl, 1 MgCl_2_, 2 CaCl_2_, 10 glucose, and 10 HEPES); (b) 3 mM HEPES solution (150 NaCl, 5 KCl, 1 MgCl_2_, 2 CaCl_2_, 10 glucose, and 3 HEPES). The pH was adjusted to 7.4 using NaOH. (c) CO_2_/bicarbonate solution (120 NaCl, 22 NaHCO_3_, 4.5 KCl, 1 MgCl_2_, 2 CaCl_2_, and 11 glucose). The pH was adjusted to 7.4 by continuous bubbling with 5% CO_2_. All solutions were in equilibrium with atmospheric O_2_ partial pressure. The reaction rate of protons and the anions is assumed to be much faster than the speed of their diffusion rate. HEPES: pKa = 7.5^64^; 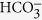: pKa1 = 6.3^65^,


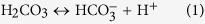


(3) The diffusion rate of molecules including H^+^, OH^−^, HEPES, HEPES^−^, 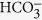, and H_2_CO_3_. The diffusion flux per unit area (*J*) was computed according to the diffusion law^44^:


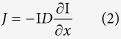


where I is the concentration of the proton, *x* is the spatial distance, and *D* is the diffusion coefficient. The diffusion coefficients used were: 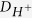 = 9.3 × 10^−9^ m^2^/s^44^; 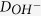 = 5.3 × 10^−9^ m^2^/s^44^; 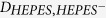 =  5 × 10^−10^ m^2^/s^64^; and 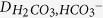 = 1.46 × 10^−9^ m^2^/s^66^.

By combining the computer simulation model with the whole-cell recording data of the proton current, we can determine the pH changes at any extracellular point once protons are released from the single cell. The detection points were varied from 10 nm to 100 nm to mimic the distance between the synapse or neuron-glia interface. Two detection points in the model are shown, as indicated by the red and green discs in Fig. 5a. The red disc was located in the centre of the neighbouring region of two cells, while the green disc was placed 10 nm from the source cell (blue spheroid), opposite to the red one.

### Reagents and data analysis

All the drugs were purchased from Sigma-Aldrich, unless stated otherwise. PcTX1 was purchased from the Peptide Institute. Results are expressed as means ± SEM. Statistical comparisons were performed using unpaired or paired *t*-tests, where *p* values < 0.05 are considered significant.

## Additional Information

**How to cite this article**: Zeng, W.-Z. *et al.* Activation of acid-sensing ion channels by localized proton transient reveals their role in proton signaling. *Sci. Rep.* **5**, 14125; doi: 10.1038/srep14125 (2015).

## Supplementary Material

Supplementary Information
