# Supplementary material for: Activation of acid-sensing ion channels by localized proton transient reveals their role in proton signaling
Source: Sci Rep. 2015 Sep 15;5:14125. doi: 10.1038/srep14125 (PMC4569896; doi:10.1038/srep14125)
Supplement: Supplementary Information [file srep14125-s1.doc]

**Supplementary Information**

**Activation of acid-sensing ion channels by localized proton transient reveals their role in proton signaling**

Wei-Zheng Zeng^1,#^, Di-Shi Liu^1,#^, Lu Liu^2^, Liang She^2^, Long-Jun Wu^3,*^, Tian-Le Xu^1,*^

^1^ *Discipline of Neuroscience,* *Department of Anatomy, Histology and Embryology, Collaborative Innovation Center for Brain Science, Shanghai Key Laboratory for Tumor Microenvironment and Inflammation, Shanghai Jiao Tong University School of Medicine, Shanghai 200025, China; ^2^ Institute of Neuroscience, Chinese Academy of Sciences, Shanghai 200031, China; ^3^ Department of Cell Biology and Neuroscience, Rutgers University, Piscataway, NJ 08854, USA*

*^#^ These authors contributed equally to this work.*

Email: [xu-happiness@shsmu.edu.cn](mailto:xu-happiness@shsmu.edu.cn) or [lwu@dls.rutgers.edu](mailto:lwu@dls.rutgers.edu)

**Inventory of Supplementary Information**

**Supplementary Figures**

Supplementary Figure S1

Supplementary Figure S2

Supplementary Figure S3

**Supplementary Figure S1**


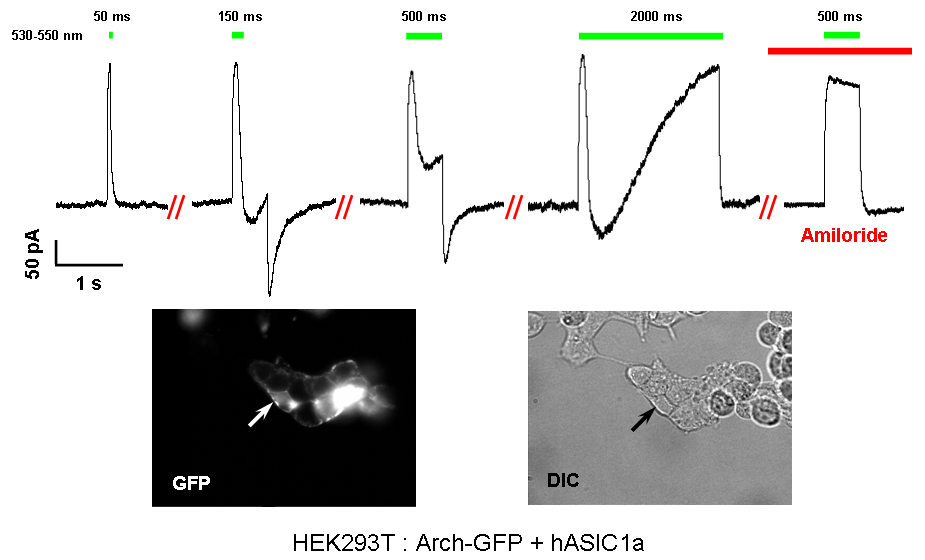


**Supplementary Figure S1. Correlation between inward current and light intensity.** HEK293T cells were cotransfected with Arch-GFP and human ASIC1a. Different light intensity was achieved by controlling the illumination time, which was powered by a high-speed shutter responding from 50 to 2000 ms. Before the end of last illumination, pan-ASICs channel blocker amiloride (100 μM) was added into the medium.

**Supplementary Figure S2**


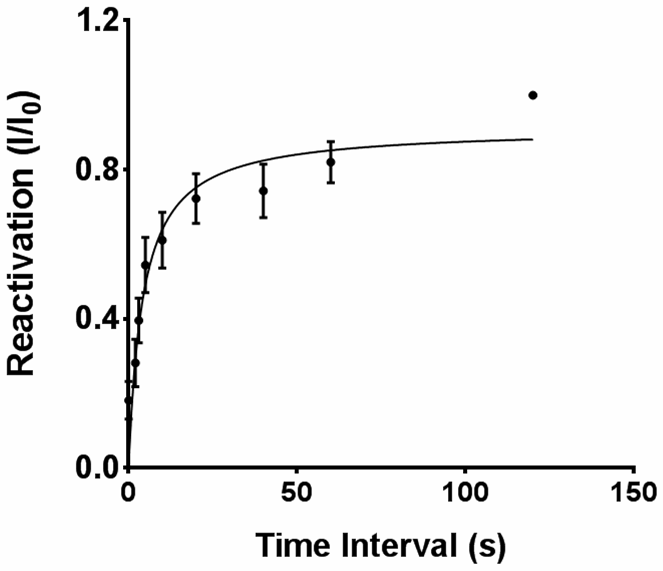


**Supplementary Figure S2. Reactivation curve of Arch-induced ASIC1a current.** Cells were cotransfected with Arch and ASIC1a. Whole-cell recordings were performed 24-48 hr after transfection. Cells were held at -60 mV and currents activated by 530-550 nm green light. For each cell, several traces are superimposed, including one control (I_0_) and several subsequent reactivated currents (I) recorded at 2, 3, 5, 10, 20, 40, 60 and 120 s after the control light stimulation. The reactivation ratios (I/I_0_) are plotted as a function of time intervals. Data are fitted with a single exponential function (τ = 8.5 s, n = 11). Error bars indicate means ± SEM.

**Supplementary Figure S3**

**
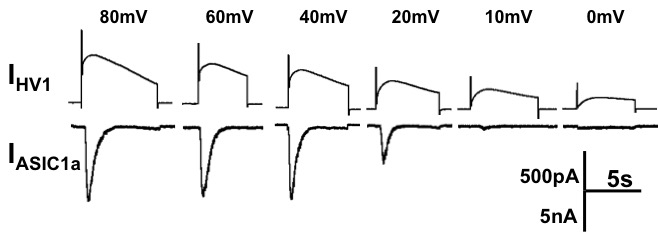
**

**Supplementary Figure S3. Voltage-dependent activation of ASIC1a by protons released through Hv1 channel in neighboring cells (Related to Figure 6).** Hv1 proton currents and ASIC1a currents in HEK293T cells expressing Hv1 and ASIC1a were recorded by two-cell sniffer patch (Similar results were obtained from other three cells). The holding potential of Hv1 expressing cells is -60 mV. Induction of Hv1 current is achieved by depolarization from -60 mV to various voltage potentials, which was indicated above. As expected, the amplitude of ASIC currents is closely correlated with that of Hv1 currents.
